# Retrospective evaluation of “Rods and Rings” pattern detected in the anti-nuclear antibody (ANA) indirect immunofluorescence (IIF) test

**DOI:** 10.3389/fimmu.2024.1359030

**Published:** 2024-06-07

**Authors:** Neşe İnal, Berke Kurumanastırlı, Tutku Taşkınoğlu, Alev Çetin Duran, Alper Togay, Fatma Mutlu Sarıgüzel, Nilgün Kaşifoğlu, Mehmet Soylu, Yavuz Doğan, Ebru Us, Zeynep Sarıbaş, Neşe Kaklıkkaya, Burçin Şener

**Affiliations:** ^1^ Department of Medical Microbiology, Hacettepe University Faculty of Medicine, Ankara, Türkiye; ^2^ Department of Medical Microbiology, Ankara Düzen Laboratories, Ankara, Türkiye; ^3^ Department of Medical Microbiology, Health Sciences University Balıkesir Atatürk City Hospital, Balıkesir, Türkiye; ^4^ Department of Medical Microbiology, Health Sciences University İzmir Tepecik Training and Research Hospital, İzmir, Türkiye; ^5^ Department of Medical Microbiology, Erciyes University Faculty of Medicine, Kayseri, Türkiye; ^6^ Department of Medical Microbiology, Eskişehir Osmangazi University Faculty of Medicine, Eskişehir, Türkiye; ^7^ Department of Medical Microbiology, Ege University Faculty of Medicine, İzmir, Türkiye; ^8^ Department of Medical Microbiology, Dokuz Eylül University Faculty of Medicine, İzmir, Türkiye; ^9^ Department of Medical Microbiology, Ankara University Faculty of Medicine, Ankara, Türkiye; ^10^ Department of Medical Microbiology, Karadeniz Technical University Faculty of Medicine, Trabzon, Türkiye

**Keywords:** anti-rods and rings (Anti-RR), HCV, anti-nuclear antibodies (ANA), indirect immunofluorescence (IIF), immunology

## Abstract

**Introduction:**

Anti-rods and rings (anti-RR) antibodies have recently been described as a cytoplasmic pattern in IIF-based screening of autoantibodies on HEp-2 cells and ICAP has named it as AC-23. It is most frequently related to drug-induced antibody generation. This study aimed to investigate the clinical significance of AC-23 positivity and its relevance to the diagnosis and/or follow-up of the associated diseases and/or drug use.

**Methods:**

A multicenter retrospective study was conducted among 10 hospitals from six different provinces in Türkiye from January 2017 to December 2021. The laboratory data and clinical information of 600 patients with positive anti-RR antibodies out of 547.558 HEp-2 IIF ANA samples were analyzed.

**Results:**

The distribution of AC-23 positive patients by year indicated a steady increase between 2017-2021. Anti-RR prevalence in post-COVID-19 period was significantly higher than that of pre-COVID-19 period (p=0.00). Concomitant ANA positivity was detected in 56.5% of patients, the most common patterns being AC-4 and AC-5 (41.1%). The most frequent pathology among the anti-RR positive patients was an autoimmune disease (19.83%); 28.57% of which had rheumatoid arthritis and 17.65% autoimmune liver disease. Among the 600 patients, 65 (10.83%) were diagnosed as hepatitis C virus (HCV) infection. Available data for 38 of the HCV patients revealed that 71.05% of them had a history of interferon alfa+ribavirin and 28.95% of them had a history of NS3/4/5A/5B polymerase inhibitor or protease inhibitor drug use. Significant increase in the rate of anti-RR positivity was observed in the post-COVID-19 period when compared to pre-COVID-19 period (p:0.00).

**Discussion:**

This is the first multicenter study in Türkiye about the clinical association of anti-RR antibodies which may be ignored during routine HEp-2 IIF testing. Pathologies other than HCV should be taken into consideration in terms of the possible role of anti-RR in autoimmune diseases and other pathologies. The preliminary data obtained in this study suggest that anti-RR antibody development might also be associated to COVID-19, supporting the several previous data related to the potential of viruses triggering the formation of autoantibodies. Large-scale prospective studies should elucidate the clinical significance of RR pattern and determine its role in patient diagnosis and follow-up.

## Introduction

Anti-nuclear antibodies (ANA) are specific antibodies that target nuclear and cytoplasmic proteins (1, 2). Indirect immunofluorescence microscopy (IIF) is considered to be the gold standard method for detecting ANA (3). The intensity and pattern of fluorescence signals detected in the nucleus and cytoplasm are evaluated for the presence of specific autoantibodies.

In the year 2005, a novel cytoplasmic pattern of ANA called “anti-rods and rings (anti-RR)”, later denoted as AC-23 by the International Consensus on ANA Patterns (ICAP) was identified in sera from hepatitis C virus infected patients who were treated with pegylated interferon plus ribavirin combination therapy (PEG-IFN/RBV) (4). Anti-RR antibodies lead to the development of fibrillary structures resembling rods (3-10 microns in length) and rings (2-5 microns in diameter). Anti-RR antibodies mainly target inosine-5’-monophosphate dehydrogenase (IMPDH) which catalyzes the rate limiting step of *de novo* guanine nucleotide synthesis (2, 5). Anti-IMPDH antibodies display cytoplasmic rods and rings pattern in IIF-HEp-2 (6).

HCV infection has a high potential to become chronic in the course of treatment and might lead to high consequence clinical presentations such as hepatocellular cancer, cirrhosis, and immunological interactions. HCV mainly induces B lymphocyte proliferation which is presumed to be the underlying mechanism for the emergence of anti-RR (7). The function and medical relevance of the anti-RR antibodies was not completely elucidated and some studies indicated that these antibodies may be stimulated by the use of some drugs such as ribavirin and IFN. The emergence of anti-RR antibodies is highly related to the duration and dosing of the anti-viral therapy (8, 9). In addition, methotrexate, azathioprine, and acyclovir were reported to be involved in the formation of drug-induced RR structures in the cells (8). Anti-RR antibodies were also associated with metabolic disorders and considered to be a possible manifestation of adaptive response associated with metabolic disorders in nonhepatitis infected patients (10). These antibodies were reported to be associated with some other chronic diseases such as hepatitis B infection, chronic renal failure, diabetes mellitus, chronic obstructive pulmonary disease, hypertension, and even in healthy individuals (2).

This study aimed to provide laboratory and clinical data about anti-RR positivity in Türkiye in a selected large-scale laboratory cohort and to evaluate the association of RR pattern in different disease settings including COVID-19 to help building data for further research.

## Method

A multicenter retrospective study was conducted among 10 hospitals from six different provinces in Türkiye from January 2017 to December 2021. In this study, demographic data, clinical diagnosis, presence of HCV infection, drugs used for HCV treatments, other drugs used for treatment of diseases, and laboratory data of patients with RR pattern in the ANA HEp-2 IIF test were obtained from the laboratory data systems of the participating centers. Nine of the participating centers were public laboratories (training and research hospitals or university hospitals) and one was a high capacity private laboratory.

The clinical data related to the patients were obtained in each center by the related author, from the hospital information management system (HIMS). The clinical diagnosis of the patients were confirmed by the use of epicrisis notes and/or patient drug reports. All the demographic and clinical data were withdrawn from the HIMS at the time of anti-RR positive result or within two months before or after the positive result. To prevent duplicated data, single results of the patients were included.

All participant laboratories performed ANA test by indirect immunofluorescence assay (IIF) with HEp-2 cells (EUROIMMUN, Lübeck, Germany), using serial dilutions commencing at 1:100. The results were observed by the fluorescence microscope (EUROStarIII Plus). The homogeneity in HEp-2 IIF testing and interpretation was achieved by the experienced and well-trained specialists in the author list. All the participants of the study group are the members of KLIMUD (Society for Clinical Microbiologists of Türkiye) Basic Immunology Study Group and follow the ICAP guidelines and KLIMUD Guide for the Laboratory Diagnosis of Autoantibodies (4, 11).

Statistical analyses were performed with IBM SPSS Statistics, version 26.0 (IBM Corp., Armonk, NY). A p-value of <0.05 was considered statistically significant. Mean, standard deviation, and minimum-maximum values ​​were determined for continuous variables. The suitability of the data for normal distribution was evaluated. If the data were normally distributed and parametric conditions were met in terms of the measured parameters, the independent sample-t test between groups was used. Since non-parametric situations occur if the data do not comply with normal distribution, both groups were evaluated with the Mann-Whitney U test. The rates of anti-RR positivities between pre- and post-COVID-19 era were compared by Chi-square test and Odds ratio (95% CI) were calculated.

This study was approved by the Hacettepe University Non-Interventional Clinical Research Ethics Board (Project No: GO 22/359, Approval No: 2022/06-48).

## Results

A total of 547.558 HEp-2 IIF ANA samples were tested in the study centers between 2017 and 2021. Repeated patient samples were excluded from the study. Among these 0.11% (600 patients) were reported positive for anti-RR antibodies. Of the 600 patients whose mean ± SD ages were 48.80 ± 19.12 (range: 1-90) years, 403 (67.17%) were females and 197 (33.83%) were males. Anti-RR antibody positivity was commonly observed in patients among the middle-aged groups (18-65 years). 29.17% of the patients applied to rheumatology clinics. Isolated anti-RR positivity was detected in 43.50% of the 600 patients. Concomitant ANA positivity was detected in 56.50% of the anti-RR positive patients, the most common patterns being AC-4 and AC-5 (**Table 1**).

**Table 1:** Demographic, clinical, and virological data in patients with anti-RR positivity.

		Number (n)	Percent (%)
Gender	**Female**	403	67.17%
	**Male**	197	32.83%
Age	**0-18**	43	7.17%
	**18-65**	413	68.83%
	**>65**	144	24.00%
Clinics Applied	**Rheumatology**	175	29.17%
	**Gastroenterology**	86	14.33%
	**Hematology**	38	6.33%
	**Internal Medicine**	70	11.67%
	**Pediatrics**	36	6.00%
	**Others**	195	32.50%
Concomitant ANA Positivity	**Negative**	261	43.50%
	**Positive**	339	56.50%
ANA titers of patients with anti-RR positivity* (56.5%)	**(+)**	49	14.45%
	**1+**	221	65.19%
	**2+**	40	11.80%
	**3+**	29	8.56%
Concomitant ANA pattern positivity (56.5%)	**AC-1**	17	5.01%
	**AC-2**	18	5.31%
	**AC-4-5**	247	72.86%
	**AC-8-9-10**	25	7.37%
	**AC-21**	3	0.88%
	**AC-25**	6	1.78%
	Others**	23	6.78%

*ANA titer of patients with anti-RR positivity means that (+): weak positivity-1/100, (1+): >1/100-<1/320, (2+): ≥1/320-<1/1000, (3+): ≥1/1000-<1/3200.

** Includes AC-11, AC-12, AC-15, AC-19, AC-24, AC-26, AC-27 patterns.

**Figure 1:**
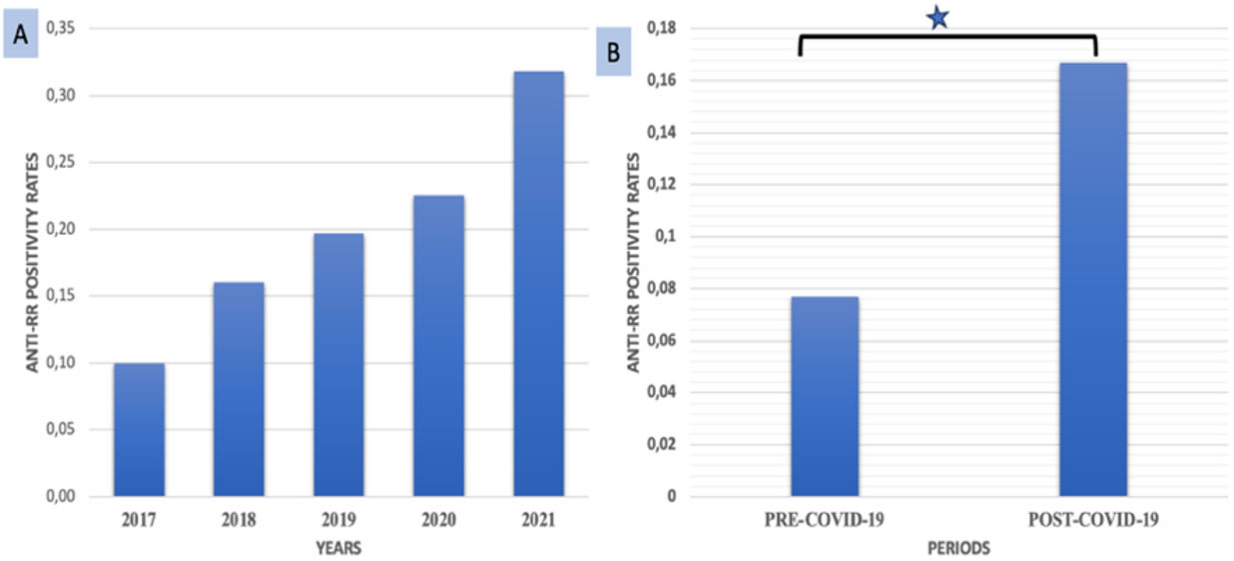
**(A)** Anti-RR rates (%) among the samples tested for ANA during 2017-2021; **(B)** Anti-RR rates in pre-COVID-19 and post-COVID-19 period (*p=0.000).

The number of HEp-2 IIF tests performed in 2017, 2018, 2019, 2020, and 2021 were 114378, 118759, 122143, 79077 and 113201, respectively. Anti-RR frequency in 2017, 2018, 2019, 2020, and 2021 were found as 0.05% (62 patients), 0.08% (97 patients), 0.10% (119 patients), 0.18% (138 patients), 0.16% (184 patients), respectively (**Figure 1**). The distribution of AC-23 positive patients by year indicated a steady increase between 2017 and 2021. While 10% of the AC-23 patients were detected in 2017, 32% were in 2021 (**Figure 1A**).

Anti-RR prevalence in pre-COVID-19 period (2017, 2018, 2019) was 0.08 while it was 0.17 in the post-COVID-19 period (2020, 2021). The difference in the rates in relation to COVID-19 was significant (**Figure 1B**). Anti-RR positivity was 2.16-fold [95% CI: 1.84 to 2.54] higher in the post-COVID period when compared to the pre-COVID period.

The disease and drug history of the anti-RR positive patients were summarized in **Table 2**. Among the 600 anti-RR antibody positive patients included, 65 (10.83%) were diagnosed as HCV, 29 (4.83%) as HBV, and 9 (1.50%) as HCV+HBV infection. Data about the anti-viral therapy of HCV patients were available for 38 patients; 27 (71.05%) of them had a history of interferon alfa+ribavirin while 11 patients (28.95%) had a history of NS3/4/5A/5B polymerase inhibitor or protease inhibitor drug use.

**Table 2:** Disease and drug history of anti-RR-positive patient.

		Number (n)	Percent (%)
**Autoimmune Diseases** **(n:119)**	SLE	12	10.08%
	RA	34	28.57%
	Autoimmune Liver Disease	20	16.81%
	Other Autoimmune Diseases	53	44.54%
**Hepatitis** **(n:94)**	HBV	29	30.85%
	HCV	65	69.15%
**Anti-viral Drug Use of HCV Patients** **(n:38)**	PEG-IFN/RBV	27	71.05%
	NS3/4/5A/5B polymerase inhibitor or protease inhibitor	11	28.95%
**Use of Other Drugs** **(n:245)**	Corticosteroid	45	18.36%
	Hydroxychloroquine	23	9.38%
	Colchicine	20	8.16%
	Immunosuppressive drugs	27	11.02%
	TNF, CD20 or cytokine inhibitors	10	4.08%
	Other drugs	120	48.9%

Autoimmune Liver Disease, including autoimmune hepatitis and one patient with primary biliary cholangitis; Immunosuppressive drugs, including methotrexate, mycophenolate, tacrolimus, cyclophosphamide, azathioprine, leflunomide, tofacitinib; Other autoimmune diseases, including undifferentiated connective tissue disease, Graves’ disease, sjogren’s syndrome, systemic scleroderma.

HBV, Hepatitis B; HCV, Hepatitis C; RA, rheumatoid arthritis; SLE, systemic lupus erythematosus; PEG-IFN/RBV, pegylated interferon plus ribavirin combination therapy.

An autoimmune pathology was defined in %19.8 (119/600) of the anti-RR positive patients. The most frequent autoimmune diseases among these 119 patients were rheumatoid arthritis (28.57%; 34 patients), and autoimmune liver disease (17.65%; 20 patients with autoimmune hepatitis (AIH) and one patient with primary biliary cholangitis). One of the patients with AIH had concomitant HCV infection, however, the patient’s anti-HCV drug treatment data was absent. The remaining autoimmune diseases defined in the anti-RR positive patients were systemic lupus erythematosus, ankylosing spondylitis, familial mediterranean fever, anti-phospholipid syndrome, Sjogren’s syndrome, myasthenia gravis, Wegener’s granulomatosis, spondyloarthritis, Behcet’s disease, multiple sclerosis, myositis, systemic sclerosis, and psoriasis. Sixty (10.00%) of the anti-RR positive patients had diabetes mellitus and 43 (7.16%) had vitamin-D deficiency.

Data in terms of drug use other than HCV-targeted drugs was available for 245 patients. Among these 18.36% (45/245) had corticosteroid use, 9.38% (23/245) hydroxychloroquine use, 8.16% (20/245) colchicine use, 11.02% (27/245) immunosuppressive drugs as methotrexate, mycophenolate, tacrolimus, cyclophosphamide, azathioprine, leflunomide, tofacitinib, 4.08% (10/245) use of TNF, or CD20 or cytokine inhibitors (**Table 2**).

The demographic and clinical data of the RR-positive patients in terms of pre- and post-COVID-19 era were given in **Table 3**. Significant difference was observed for the age and HCV disease variables. It was observed that the age of the anti-RR positive patients in the post-COVID-19 era were less than that of pre-COVID period (p=0.002). The other striking finding was that among these anti-RR positive patients, the rate of HCV positive patients was less in the post-COVID than the pre-COVID era.

**Table 3:** The relationship between anti-RR positive patients and underlying diseases in the pre-COVID and post-COVID period.

	Pre-COVID	Post-COVID	Chi-square	p	OR (Post/Pre) [%95 GA]
	n (%)	n (%)			
**Age (Mean ± SD)**	51.49 ± 18.50	46.61 ± 19.38		**0.002**	
**Gender**			0.000	0.995	
Female	219 (67.2)	184 (67.2)			
Male	107 (32.8)	90 (32.8)			
Acute Infection*			0.858	0,354	1.336 [0.722; 2.472]
Positive	18 (6.6)	28 (8.6)			
Negative	256 (93.4)	298 (91.4)			
**Autoimmune Diseases**			0.967	0,325	1.225 [0.817; 1.836]
Positive	49 (18.2)	70 (21.5)			
Negative	225 (81.8)	256 (78.5)			
**CNS Diseases**			0.433	0,510	1.233 [0.661; 2.3]
Positive	18 (6.6)	26 (8)			
Negative	256 (93.4)	300 (92)			
**Pulmonary Diseases**			0.873	0,350	1.372 [0.705; 2.671]
Positive	15 (5.5)	24 (7.4)			
Negative	259 (94.5)	302 (92.6)			
**Metabolic Diseases**			0	0,990	1.003 [0.666; 1.51]
Positive	52 (19)	62 (19)			
Negative	222 (81)	264 (81)			
**CVS Diseases**			0.269	0,604	0.876 [0.532; 1.443]
Positive	34 (12.4)	36 (11)			
Negative	240 (87.6)	290 (89)			
**Genitourinary Diseases**			0.382	0,536	1.212 [0.658; 2.231]
Positive	19 (6.9)	27 (8.3)			
Negative	255 (93.1)	299 (91.7)			
**Gastrointestinal Disorders**			0.023	0,880	0.965 [0.608; 1.532]
Positive	39 (14.2)	45 (13.8)			
Negative	235 (85.8)	281 (86.2)			
**Diagnosis of HCV**			8.905	**0,003**	0.452 [0.265; 0.769]
Positive	41 (15)	24 (7.4)			
Negative	233 (85)	302 (92.6)			
**Diagnosis of HBV**			3.304	0,069	0.497 [0.23; 1.071]
Positive	18 (6.6)	11 (3.4)			
Negative	256 (93.4)	315 (96.6)			

CNS, Central nervous system; CVS, Cardiovascular system.

*: Upper/lower respiratory tract infection, urinary tract infection, gastroenteritis, endocarditis, optic neuritis, EBV, CMV, Varicella zoster virus, Herpes simplex virus infection.

Bold numbers indicate *p<0.05.

## Discussion

It is considered that major nuclear and cytoplasmic HEp-2 IIF patterns are clinically relevant. ICAP has established a nomenclature for the harmonization of HEp-2 IIF patterns reporting. According to the ICAP, nuclear patterns homogenous (AC-1), dense fine speckled (DFS; AC-2), centromere (AC-3), speckled (AC-4, 5), discrete dots (AC-6, 7), nucleolar (AC-10, 11, 12) and cytoplasmic patterns fibrillary (AC-15, 16, 17), speckled (AC-18, 19, 20), reticular/mitochondria-like (AC-21), polar/Golgi-like (AC-22) and rods and rings (AC-23) are the competent-level patterns (4). According to a global survey conducted in 2020, the rods and rings pattern (AC-23) was the least used pattern (63% of the laboratories) in reporting ANA results. AC-23 has been found to be more used by expert-level laboratories than by competent-level laboratories. The related survey also revealed that AC-23, together with AC-22 had the lowest score for clinical relevance by the laboratory professionals (12). The Basic Immunology Study Group working under the Society for Clinical Microbiologists of Türkiye is aimed to promote harmonization of HEp-2 IIF patterns among the autoimmune disease diagnostic laboratories in Türkiye since 2011. During the evaluation of our routine HEp-2 IIF results it was noticed that there had been an increase in the rate of AC-23 pattern recognition and reporting in many laboratories in our country especially following the COVID-19 pandemic. Thus, a retrospective large-scale study was planned to evaluate the AC-23 pattern frequency and the related demographic and clinical features in ten participating centers with selected capacity and experience for ANA diagnostics.

It has been previously mentioned that the AC-23 pattern is only detectable in certain HEp-2 slides (13). All the participating centers in this study used Euroimmun HEp-2 cells which enabled the recognition of AC-23 pattern and besides all the participants had been trained and experienced for HEp-2 IIF patterns and AC nomenclature. Considering the current literature this is the largest ANA HEp-2 IIF series evaluated for the presence of AC-23 in a selected large-scale laboratory cohort. The results of the current study revealed that among a collection of 547.558 samples investigated for ANA HEp-2 IIF analysis, 0.11% of the patients were positive for the anti-rods and rings antibodies. Anti-RR prevalence has been known to exhibit geographical variation. There are few studies in the literature from Türkiye about anti-RR prevalence. In a retrospective evaluation of 41.921 serum samples with HEp-2 IIF conducted in a tertiary healthcare hospital in Izmir, anti-RR positivity was detected in only 0.01% of the samples, which was exceptionally lower than the anti-RR antibody positivity rates reported in the literature (14) and in the current study. The difference between the results of the current study and the study from Izmir, Türkiye, might be attributed to the different patient populations, the multi-center nature of the current study and the different time periods. Zhang reported anti-RR prevalence as 0.10% in western China in 2016-2018 period (2). However, Meng et al. reported anti-RR frequencies of 0.16% and 0.23% in two different large hospitals in China between 2018 and 2020 (15). Considering these results, there is a wide variety in the prevalence of RR positive patients in different locations. It is also a matter of concern that RR pattern cannot be detected by the reagents of some HEp-2 manufacturers (16). This may impact the detection of RR pattern in different settings which may also have an impact to correlate RR pattern with different disease conditions.

The results of the current study showed that anti-RR prevalence was significantly higher in the post-COVID-19 period than the pre-COVID-19 period. The increased rate of anti-RR positivity in the post-COVID era was one of the significant findings of the current study. The younger age of the anti-RR positive patients in the post-COVID-19 era in comparison to the pre-COVID era, might emphasize the possible autoantibody triggering potential of COVID-19. It is well-known that autoimmunity is more related to increasing age, however, our results indicated that anti-RR positive patients were younger following the COVID-19 period. The lower rate of HCV positive patients in the post-COVID era was another striking result of our study. This might raise the hypothesis that anti-RR antibody development is not only limited to PEG-IFN/RBV treated HCV patients, whereas other viral diseases such as COVID-19 as observed in our study, could have induced the generation of anti-RR.

The association of autoimmune and inflammatory pathologies with infectious diseases is a well-known issue. Following the COVID-19 pandemic and the global vaccination efforts, there have been several reports of increasing autoimmune conditions (17, 18). During 2020-2021 the members of our study group realized an increase in reporting AC-23 pattern in the HEp-2 IIF reports. Possible reasons for this increase might include the development of autoantibodies due to COVID-19 infection and/or vaccine or increased recognition and reporting by the laboratory specialists. Considering higher anti-RR positivity rates of post-COVID-19 period as opposed to pre-COVID-19 period, our results raised the question whether COVID-19 might be bound up with immune stimulation/dysregulation and rise in autoimmune antibodies. Owing to the retrospective nature of the current study a causal relation between COVID-19 disease and/or vaccination and the development of autoantibodies, particularly anti-RR, was not investigated. However, the significant increase in the rate of anti-RR positivity during 2020-2021 may provide evidence for planning future studies to investigate the molecular mechanisms of the related pathology. T cells are usually hyperactivated during the course of SARS-CoV-2 infection (19). Increased proliferation of activated T cells in COVID-19 disease might have induced IMPDH hyperactivation and this may further lead to the production of RR structures.

The RR pattern has been previously related to the prior use of interferon-α plus ribavirin combination therapy in HCV infected patients, suggesting that anti-RR are drug-induced autoantibodies (5, 16, 20). 20%-40% of HCV positive patients who are on pegylated interferon and ribavirin therapy usually develop anti-RR antibodies within 6 months of therapy (11). The current study showed that 10.83% of the AC-23 positive patients were diagnosed as HCV, 4.83% as HBV, and 1.50% as HCV-HBV co-infection. Data about the anti-viral therapy of HCV patients were available for 38 patients; 71.05% of them had a history of interferon alfa+ribavirin while 28.95% of them had a history of NS3/4/5A/5B polymerase inhibitor or protease inhibitor drug use. Stinton et al. reported about 5% anti-RR positivity in a cohort of 315 HCV patients in 2013 and concluded that anti-RR positive patients were significantly more likely than anti-RR negative cases to have been treated with IFN-based therapy (20). Climent et al., in their four-year retrospective study conducted in Spain, reported anti-RR positivity in 0.30% of the serum samples sent for ANA IIF analysis, and among these 87 patients, 73 were HCV positive (21). On the other hand, Afsharzadeh et al. reported a single case with anti-RR positivity in a cohort of 120 HCV patients in Iran and this case also previously had received IFN/ribavirin antiviral therapy (22). Peker et al. reported an overall 15.16% anti-RR positivity in a cohort of HCV patients that received pegylated IFN and/or ribavirin. They concluded that RR formation was significantly higher in treatment regimens with pegylated interferon and ribavirin than in treatment regimens without pegylated interferon and ribavirin (23). The results of the current study supported the evidence for the relation of RR development with IFN/ribavirin therapy.

Some researchers have reported that the presence of anti-RR is closely related to the relapse of the virus. Covini et al. found anti-RR positivity rates to be 33% in HCV patients who did not respond to treatment or had virus relapse and 11% in patient groups in which the virus was eliminated (24). Owing to the retrospective nature of the current study, data about the progress of HCV disease and treatment was insufficient for a reliable analysis.

The RR pattern has also been reported rarely in non-HCV infected individuals, including patients with systemic autoimmune diseases, patients under treatment with mycophenolic acid, methotrexate, azathioprine or acyclovir, and although very rarely in healthy people in lower titers (8, 16, 21, 24). Treatment with PEG-IFN and ribavirin have been shown to stimulate anti-RR formation. Methotrexate, azathioprine, and acyclovir were also reported to be involved in the formation of drug-induced RR structures in the cells (8). Keppeke emphasized that ribavirin-treated HCV patients showed higher percentage of anti-RR positivity than autoimmune patients treated with the anti-RR inducing immunosuppressant drugs. They also found that adefovir, entecavir, tenofovir, and lamivudine did not induce RR structures in-vitro (8). In our study, we detected RR pattern also in 28.95% of the patients with HCV treatment history of NS3/4/5A/5B polymerase inhibitor or protease inhibitor drug use. Although the structure of the current study was not appropriate to reach to a conclusion that other antiviral drugs may also induce RR formation, it provided valuable data for further research. The evaluation of anti-RR inducing drug use in our study population revealed the use of corticosteroids, hydroxychloroquine, colchicine, and immunosuppressive drugs including methotrexate, mycophenolate, tacrolimus, cyclophosphamide, azathioprine, TNF/CD20/cytokine inhibitors. It has been shown in-vitro that mycophenolic acid, which is another IMPDH inhibitor, like ribavirin, induced RR in a high percentage of cultured cells (8). However, specific information about the potential RR inducing roles of other drugs is still lacking. In our setting with a higher number of patients with autoimmune diseases, it was not possible to correlate RR presence with the immune dysregulation present in the study population and/or the use of the listed drugs. The current data supports the view that RR production seems to be a result of an immunologic tolerance breakdown followed by a complex pathway of autoantibody production.

An autoimmune pathology was defined in about 20% of the RR positive patients in the current study. In our cohort the most frequent autoimmune diseases with RR positivity were rheumatoid arthritis and autoimmune hepatitis AIH). The highest concomitant HEp-2 patterns in RR patients were the AC-4/5 nucleus speckled pattern which is mostly associated with connective tissue diseases. Meng et al. also reported higher presence of RR in patients with autoimmune diseases and concluded that autoantibodies were much more common in connective tissue diseases than that in other diseases. They interestingly noted the highest prevalence in patients with AIH and primary biliary cholangitis (PBC) (15). RR structures were also defined by Assandri in 2019 in a case with PBC (25). Autoimmune hepatitis was the second most frequent autoimmune pathology also in our cohort. Thus, our data also supported the finding that RR antibodies might be related to autoimmune diseases particularly involving the liver. In contrast to some of the previous studies relating the presence of RR antibodies vastly with IFN/ribavirin treatment and considering them as drug-induced autoantibodies (5, 8), here we report evidence for their presence also in some autoimmune diseases.

Although few in number, there are reports in the current literature about the presence of RR antibodies in several other diseases than HCV. RR pattern was also defined in patients with renal diseases, chronic obstructive pulmonary disease, hematologic disorders, and metabolic diseases (2, 10, 26). The results of the current study supported this finding in that AC-23 was observed also in patients with HBV infection, SLE, Sjogren syndrome, rheumatoid arthritis, and metabolic diseases. In the light of current information, it is obvious that the RR pattern is not restricted to HCV and related antiviral treatment, but rather it seems to be a consequence of an immune dysregulation. With the accumulation of data in relation to RR pattern and specific clinical conditions together with basic research about the molecular pathophysiology of these antibodies, their role as biomarkers of disease or their prognostic role will be better understood.

The absence of clinical information for all the patients is recognized as the main limitation of this study. The most important reason for this limitation is the retrospective nature of the study. Likewise, the data of anti-RR+ and anti-RR-ve patient subgroups could not be compared for different demographic and clinical variables. However, the result of this study contributes to the data related to anti-RR positivity in the clinical situations other than PEG-IFN/RBV treated HCV patients and also is the first study which emphasized the possible role of COVID-19 on anti-RR development. Larger-scale prospective studies should elucidate the clinical significance of HEp-2 IIF RR pattern and determine its role in patient diagnosis and follow-up.

## Conclusion

To our knowledge, the significance of anti-RR positivity in the clinical setting and its impact on diagnosis and/or prognosis are in its infancy. The current study emphasized that anti-RR positivity is not only related to anti-viral drug use in HCV patients. The clinical significance of anti-RR should be investigated in systemic autoimmune diseases which were the most emerging pathologies detected in the current study. The most striking result of our study was the increasing number of anti-RR following COVID-19 which raised the question of whether immune dysregulation during COVID-19 infection or vaccination has led to this increase. It is crucial for clinicians to recognize the possible autoimmune manifestations of COVID-19 to take action in the post-COVID-19 period. To meet the need for a better understanding of the clinical relevance of anti-RR, besides conducting large-scale prospective studies and basic research in that field, AC-23 pattern should not be overlooked while evaluating HEp-2 IIF patterns in routine laboratory practice.

## Data availability statement

The datasets presented in this study can be found in online repositories. The names of the repository/repositories and accession number(s) can be found in the article/Supplementary Material.

## Ethics statement

The studies involving humans were approved by This study was approved by the Hacettepe University Non-Interventional Clinical Research Ethics Board (Project No: GO 22/359, Approval No: 2022/06-48). The studies were conducted in accordance with the local legislation and institutional requirements. Written informed consent for participation in this study was provided by the participants’ legal guardians/next of kin.

## Author contributions

Nİ: Formal analysis, Resources, Software, Writing – original draft, Writing – review & editing. BK: Data curation, Formal analysis, Project administration, Software, Writing – original draft, Writing – review & editing. TT: Investigation, Writing – original draft, Writing – review & editing. AD: Investigation, Writing – original draft, Writing – review & editing. AT: Writing – original draft, Writing – review & editing. FS: Writing – original draft, Writing – review & editing. NK: Writing – original draft, Writing – review & editing. MS: Writing – original draft, Writing – review & editing. YD: Writing – original draft, Writing – review & editing. EU: Writing – original draft, Writing – review & editing. ZS: Writing – original draft, Writing – review & editing. NK: Writing – original draft, Writing – review & editing. BŞ: Conceptualization, Data curation, Formal analysis, Investigation, Resources, Software, Supervision, Validation, Writing – original draft, Writing – review & editing.
